# Development and external validation of a diagnostic model for differentiating major depressive disorder from bipolar disorder

**DOI:** 10.1186/s12888-026-07844-1

**Published:** 2026-01-28

**Authors:** Hongxin Zheng, Xialong Cheng, Wenxin Gan, Shuyu Duan, Yizi Liu, Kun Li, Chen Su, Chenxi Xu, Yongcan Zhou, Wenwei Zhang, Runbo Wu, Yu Xie

**Affiliations:** 1 School of Educational Science, Anhui Normal University, Wuhu, China; 2 Affiliated Psychological Hospital of Anhui Medical University, Anhui Mental Health Center, Hefei Fourth People’s Hospital, Hefei, China; 3 Tongde Hospital of Zhejiang Province, Hangzhou, China; 4 China Telecom Corporation Limited Anhui Branch, Hefei, China

**Keywords:** Bipolar disorder, Major depressive disorder, Machine learning, SHAP, External validation

## Abstract

**Background:**

Misdiagnosing bipolar disorder (BD) as major depressive disorder (MDD) can lead to poor treatment outcomes. This study aims to develop and validate a machine learning-based model to effectively differentiate between MDD and BD using electronic medical record (EMR) data.

**Methods:**

This retrospective study enrolled 584 patients with BD and 1,179 patients with MDD from two medical centers between January 2022 and August 2024. Feature selection was performed using both Least Absolute Shrinkage and Selection Operator (LASSO) regression and the Boruta algorithm. Six machine learning (ML) algorithms were used to construct the model. SHapley Additive exPlanations (SHAP) analysis was conducted to improve model interpretability.

**Results:**

Among the six machine learning models constructed based on these features, the RF model demonstrated the best overall performance, achieving the highest (AUC = 0.863) in the internal validation set and also showing moderate discriminative ability (AUC = 0.710) in the external validation set. SHAP analysis identified illness duration, creatine kinase, and age of onset as the most important predictive features.

**Conclusions:**

The 7-predictor RF model demonstrated moderate discriminative performance, showing potential as an auxiliary decision support tool for distinguishing hospitalized BD from MDD patients. However, the reliance on the International Classification of Diseases, 10th Revision (ICD-10) criteria may result in the misclassification of latent BD, thereby limiting the model’s accuracy and generalizability. Furthermore, future work should validate the model’s generalization ability in multi-center samples and develop intuitive decision support tools to enhance clinical utility.

**Clinical trial registration:**

Not applicable. This is a retrospective observational study that does not involve any clinical intervention; therefore, clinical trial registration was not required.

**Supplementary Information:**

The online version contains supplementary material available at 10.1186/s12888-026-07844-1.

## Introduction

Bipolar disorder (BD), as a recurrent mood disorder, affects approximately 2% of the global population, and its core features include extreme fluctuations in mood states, ranging from manic periods of extreme euphoria and activation to depressive periods of low mood, energy depletion, and despair [1]. Patients with BD usually experience their first episode in adolescence or early youth, and its chronic course is characterised by high morbidity and mortality [2]. This disease has severely affected social functioning and quality of life in young people and working-age groups [2]. Established research suggests that targeted interventions can help prevent people with BD from suffering potentially irreversible physical or psychological damage, further preventing the development of mood disorders [3]. However, correctly diagnosing BD is a difficult task, and related studies have observed that BD is often misdiagnosed as major depressive disorder (MDD). Such misdiagnosis may lead to inappropriate treatment strategies and, consequently, poor outcomes [4].

BD and MDD share somewhat similar clinical symptoms, especially during the depressive phases, where differential diagnosis is extremely challenging [5]. Depressive episodes are frequently the first symptoms that appear in the clinical course of BD and recur through the life of the patient, being a great burden for both the patient and their family [6]. When depressive episodes appear clinically, the disease presentation is practically the same as that of MDD. Therefore, clinicians often face challenges in differentiating MDD from BD [7]. Previous studies have shown that up to 69% of patients with bipolar disorder face misdiagnosis at initial diagnosis and are most often incorrectly diagnosed as MDD [8]. It is worth noting that about 7.6% to 12.1% of newly diagnosed MDD patients will be corrected to BD in the subsequent course [9]. Such diagnostic deviation may prevent patients with underlying BD from receiving effective treatment [10, 11].

Furthermore, given the multiple causes of BD, there are no clear biomarkers [12], and there is a shortage of easily accessible and universal diagnostic tools for the detection of BD [13]. Thus, the pressing requirement is the development of an admission screening tool that can distinguish between MDD and BD.

The increasing popularity of machine learning (ML) as a method for data processing and classification in clinical practice across multiple clinical specialties creates a possibility of employing computer techniques to detect BD [14]. Moreover, utilizing SHapley Additive explanations (SHAP) values is an evident, straightforward way of explaining the effects of different features on the model’s prediction results, thus overcoming the problem of transparency in ML models [15, 16].

Research using machine learning (ML) to distinguish MDD from BD is growing. However, a recent review of AI techniques in psychiatry pointed out that the field generally faces problems such as insufficient external validation and limited generalization ability, and these limitations seriously hinder the clinical application of machine learning models [17]. Machine learning studies using electronic medical record (EMR) data to differentiate MDD from BD are subject to the same limitations.

For example, Zhu et al. developed a diagnostic model to distinguish BD from MDD using the XGBoost algorithm based on the electronic medical record (EMR) data of 16,311 patients, and achieved an AUC value of 0.838 on single-center data [18]. Similarly, Huang et al. constructed a five-variable Logistic Regression (LR) model using EMR data from 721 patients, achieving an AUC of 0.858 [19]. However, these studies are all based on a single dataset and lack rigorous external validation to assess the generalization ability of the model.

This study developed and validated ML models for distinguishing MDD from BD using EMR data from two independent medical centers. Least absolute shrinkage and selection operator (LASSO) regression and Boruta algorithm were used for feature selection, and six ML models were constructed based on selected features. Internal validation and external testing were conducted to evaluate the performance and generalization ability of the model, and SHAP analysis was used to improve the interpretability of the model. This ML method, which has been rigorously externally validated, is helpful to identify and understand relevant predictors, and can also provide auxiliary evidence for clinicians to differentiate MDD from BD.

## Methods

### Data collection

This study retrospectively analyzed inpatients with MDD and BD who were admitted to the Fourth People’s Hospital of Hefei, Anhui Province, and Tongde Hospital of Zhejiang Province from January 2022 to August 2024. Inclusion criteria: (1) Patients with BD and MDD identified according to the diagnostic criteria of the International Statistical Classification of Mental Disorders (ICD-10); (2) Routine blood and biochemical tests completed the day after admission. Exclusion criteria: (1) Patients with co-morbid other mental illnesses; (2) Patients with serious physical illnesses; (3) Those with > 30% missing values for the primary measurements.

The project was approved by the Medical Research Ethics Committee of the Fourth People’s Hospital of Hefei (No. HFSY-IRB-YJ-KYXM-CXL [2023-034-001]), and all data carrying patient identification information were de-tagged.

Data were collected using EMR systems. The model development and internal validation cohort consisted of 991 MDD patients and 423 BD patients from the Fourth People’s Hospital of Hefei, Anhui Province. For external validation, an additional 188 MDD patients and 161 BD patients were included from Tongde Hospital of Zhejiang Province.

### Definition of outcome

The outcome measure for this study was defined as the primary discharge diagnosis for each subject, which was standardized and recorded using ICD-10 codes. The discharge diagnosis was determined by professional psychiatrists based on structured (e.g., Mini-International Neuropsychiatric Interview (MINI)) and semi-structured (e.g., Structured Clinical Interview for DSM (SCID)) diagnostic interviews, and repeatedly confirmed during hospitalization [20].

### Predictive variables

The EMR system includes sociodemographic variables, clinical variables, and physiological and biochemical variables. This study initially selected 34 variables from the EMR system, including sociodemographic variables (gender, age, marital status), clinical variables (age of onset (AOO), illness duration, family history), and physiological and biochemical indicators (triiodothyronine (T3), thyroxine (T4), thyroid-stimulating hormone (TSH), free triiodothyronine (FT3), free thyroxine (FT4), total bilirubin, direct bilirubin, albumin, alanine aminotransferase (ALT), aspartate aminotransferase (AST), gamma-glutamyl transferase (GGT), total bile acids (TBA), total cholesterol, high-density lipoprotein (HDL), triglycerides, apolipoprotein A1 (ApoA1), apolipoprotein B (ApoB), blood urea nitrogen (BUN), creatinine, uric acid (UA), potassium, sodium, chloride, calcium, creatine kinase (CK), glucose, magnesium, serum inorganic phosphorus (serum Pi)).

### Feature selection

The development cohort (*n* = 1,414) was first divided by 7:3 stratified randomization to obtain the training set (*n* = 990) and the internal test set (*n* = 424). To prevent data leakage, median imputation was performed only on the training set (*n* = 990). LASSO regression and Boruta algorithm were used for feature selection based on the training set data. The external validation cohort (*n* = 349) remained completely independent from the internal test set throughout the process and did not participate in any imputation, feature selection, or model training steps.

LASSO regression was performed for variable selection using the glmnet package in R. The L1 regularization term was introduced to minimize the binomial deviance, and the optimal regularization parameter (*λ*) was determined using 10-fold cross-validation. The training set was randomly divided into 10 subsets, with 9 subsets used for training and 1 subset was used for validation in each round. Finally, the *λ* value with the best validation performance was selected to improve the model generalization ability.

The Boruta algorithm is a feature selection method based on random forest, which helps to screen key features by comparing the importance of original features with randomly generated shadow attributes. In this study, we implemented this method using the Boruta package in R, setting the maximum number of runs to 500 and the *P*-value to 0.01.

The final set of predictors was determined by the intersection of variables selected by both the LASSO regression and the Boruta algorithm. To assess for multicollinearity among these final predictors, we calculated the Variance Inflation Factor (VIF) for each variable and computed a Pearson correlation matrix.

### ML model construction and evaluation

Six ML algorithms – Random Forest (RF), LR, LightGBM (LGB), Support Vector Machine (SVM), K-Nearest Neighbors (KNN), and XGBoost (XGB) – were used to construct the diagnostic model.

In the development cohort, we used scikit-learn’s train_test_split function for stratified train-test splitting in a 7:3 ratio. Missing values (≤ 5% per feature) were imputed using the median strategy, where the median of each feature was calculated from the training set and applied to both the training and internal test sets. To address class imbalance and prevent data leakage, we fitted StandardScaler for data standardization and SMOTE for oversampling on the training set only. The training set was processed with both SMOTE oversampling and standardization, while the internal test set was only standardized using the same StandardScaler parameters fitted on the training data.

To optimize model performance, we employed Grid Search with 10-fold stratified cross-validation (GridSearchCV) within the training set. The hyperparameter search space and final optimized parameters for each model are detailed in Supplementary Table S1. The objective function for optimization was the area under the ROC curve (AUC). The best-performing hyperparameters identified through cross-validation were used to train the final models on the entire training set.

Six ML models were trained on the processed training data and evaluated on the internal test set. We calculated accuracy (ACC), sensitivity (SEN), specificity (SPE), positive predictive value (PPV), negative predictive value (NPV), and Balanced Accuracy (BAC). To assess the stability of model performance, 95% confidence intervals (95% CI) for AUC were calculated using bootstrapping with 1,000 resamples. Model discrimination was assessed using the area under the Receiver Operating Characteristic (ROC) curve, and clinical utility was evaluated through Decision Curve Analysis (DCA).

### External validation of ML models

To evaluate the performance and generalizability of the six trained models, we tested them on an independent external validation dataset. The external data were preprocessed using the same standardization parameters (StandardScaler) that were fitted on the development cohort’s training set, ensuring consistent data transformation without data leakage. We calculated each model’s ACC, SEN, SPE, PPV, NPV, and BAC on the external validation set, with 95% CI for AUC. Model discrimination was assessed using the AUC, and clinical utility was evaluated through DCA.

### Model explanation

To understand the predictive behavior of our final RF model (selected based on its highest AUC in the internal validation set), we conducted a comprehensive SHAP analysis on the independent external validation set. The SHAP method provides an intuitive way to understand the impact of different features on model predictions using Shapley values from cooperative game theory [21]. We employed multiple SHAP visualization techniques: beeswarm plots to show feature importance and value distributions, waterfall plots to illustrate individual prediction explanations, and summary plots to demonstrate overall feature contributions to model decisions.

### Statistical analysis

Statistical analyses were performed using Python 3.12 and R 4.4.2. The *χ²* test was used for categorical variables, and the t-test for independent samples was applied to continuous variables. To account for multiple comparisons, the Benjamini-Hochberg False Discovery Rate correction was applied, with adjusted *P*-values < 0.05 considered statistically significant. The test level was set at *α* = 0.05 for a two-sided test. Differences were considered statistically significant at a *P*-value < 0.05. Missing continuous data (≤ 5% per feature) were handled with median imputation, replacing missing values with the median of observed values for that feature.

## Results

### Descriptive analysis of features

This study enrolled a total of 1,763 inpatients from two distinct cohorts, which were designated for model training and external validation. The development cohort (*n* = 1,414) was composed of 859 females (60.7%) and 555 males (39.3%), with a diagnostic distribution of 991 MDD (70.1%) and 423 BD (29.9%) cases. The independent external validation cohort (*n* = 349) included 232 females (66.5%) and 117 males (33.5%), with 188 MDD (53.9%) and 161 BD cases (46.1%).

Table 1 compares the baseline characteristics of the development and external validation cohorts. The differences between MDD and BD patients within each of these groups are detailed in the supplementary materials (Supplementary Tables S2 and S3).

**Table 1:** Baseline characteristics of the development and external validation cohorts

Variable	Development Cohort (*n* = 1414)	External Validation (*n* = 349)	(χ²/t)	*P*	Adjusted *P*
Group			32.501	< 0.001	< 0.001
MDD	991 (70.1%)	188 (53.9%)			
BD	423 (29.9%)	161 (46.1%)			
Gender			3.652	0.056	0.086
Female	859 (60.7%)	232 (66.5%)			
Male	555 (39.3%)	117 (33.5%)			
Marital Status			103.570	< 0.001	< 0.001
Unmarried	455 (32.2%)	216 (61.9%)			
Married	959 (67.8%)	133 (38.1%)			
Family History			0.007	0.932	0.937
No	1301 (92.0%)	320 (91.7%)			
Yes	113 (8.0%)	29 (8.3%)			
Age	39.803 ± 14.493	30.169 ± 13.743	-11.234	< 0.001	< 0.001
Age of Onset	32.986 ± 14.547	24.905 ± 11.776	-9.628	< 0.001	< 0.001
Illness Duration	7.201 ± 8.350	5.113 ± 6.337	-4.371	< 0.001	< 0.001
T3	1.410 ± 0.465	1.429 ± 0.376	0.700	0.484	0.564
T4	55.328 ± 50.975	114.912 ± 40.897	20.157	< 0.001	< 0.001
TSH	2.206 ± 1.886	1.915 ± 1.836	-2.588	0.010	0.021
FT3	4.152 ± 1.296	5.029 ± 0.930	11.905	< 0.001	< 0.001
FT4	8.573 ± 7.548	11.527 ± 2.982	7.175	< 0.001	< 0.001
Total Bilirubin	11.625 ± 6.619	12.360 ± 5.960	1.892	0.059	0.082
Direct Bilirubin	4.710 ± 2.645	2.318 ± 1.164	-16.476	< 0.001	< 0.001
Albumin	42.512 ± 4.001	40.989 ± 4.129	6.319	< 0.001	< 0.001
ALT	21.773 ± 23.006	19.633 ± 17.388	-1.623	0.105	0.141
AST	19.935 ± 15.020	22.294 ± 16.326	2.575	0.010	0.019
GGT	25.151 ± 27.655	23.552 ± 22.543	-0.994	0.321	0.387
TBA	4.086 ± 3.526	4.801 ± 7.247	2.613	0.009	0.019
Total Cholesterol	4.328 ± 1.016	4.347 ± 0.987	0.304	0.761	0.807
HDL	1.281 ± 0.418	1.234 ± 0.332	-1.894	0.058	0.082
Triglycerides	1.385 ± 0.996	1.243 ± 0.781	-2.429	0.015	0.027
ApoA1	1.281 ± 0.463	1.321 ± 0.287	1.507	0.132	0.171
ApoB	0.809 ± 0.406	0.829 ± 0.244	0.847	0.397	0.463
BUN	4.426 ± 1.550	4.515 ± 5.352	0.539	0.590	0.646
Creatinine	62.950 ± 16.830	63.029 ± 15.919	0.079	0.937	0.937
Uric Acid	308.692 ± 105.191	334.391 ± 114.859	4.012	< 0.001	< 0.001
Potassium	3.915 ± 0.373	3.802 ± 0.314	-5.188	< 0.001	< 0.001
Sodium	141.678 ± 3.063	138.827 ± 2.988	-15.557	< 0.001	< 0.001
Chloride	105.317 ± 2.833	104.739 ± 2.208	-3.526	< 0.001	0.001
Calcium	2.323 ± 0.207	2.296 ± 0.152	-2.271	0.023	0.037
CK	182.158 ± 495.229	220.012 ± 678.616	1.169	0.243	0.303
Glucose	5.098 ± 1.339	4.916 ± 1.018	-2.330	0.020	0.033
Magnesium	0.899 ± 0.112	0.881 ± 0.078	-2.675	0.008	0.016
Serum Pi	1.201 ± 0.173	1.340 ± 0.212	12.652	< 0.001	< 0.001

NOTE: T3: Triiodothyronine; T4: Tetraiodothyronine; TSH: Thyroid Stimulating Hormone; FT3: Free Triiodothyronine; FT4: Free Thyroxine; ALT: Alanine Aminotransferase; AST: Aspartate Aminotransferase; GGT: Gamma-Glutamyl Transferase; TBA: Total Bile Acid; HDL: High Density Lipoprotein; ApoA1: Apolipoprotein A1; ApoB: Apolipoprotein B; BUN: Blood Urea Nitrogen; CK: Creatine Kinase; Serum Pi: Serum Inorganic Phosphate; Adjusted *P*: P-values were adjusted for multiple comparisons using the Benjamini-Hochberg False Discovery Rate (FDR) method

### Feature selection

For the training set, feature selection was performed using two distinct methods. LASSO regression, applying the one-standard-error rule with an optimal λ value of 0.03734609, identified 7 predictive features: gender, AOO, illness duration, AST, BUN, UA, and CK. In parallel, the Boruta algorithm pinpointed 21 important features: gender, age, marital status, AOO, illness duration, TSH, FT3, FT4, total bilirubin, direct bilirubin, ALT, AST, HDL, triglycerides, BUN, creatinine, UA, chloride, CK, glucose, and serum Pi. By taking the intersection of these two sets, the final set of 7 candidate features for this cohort was determined: gender, AOO, illness duration, AST, BUN, UA, and CK.

**Fig. 1:**
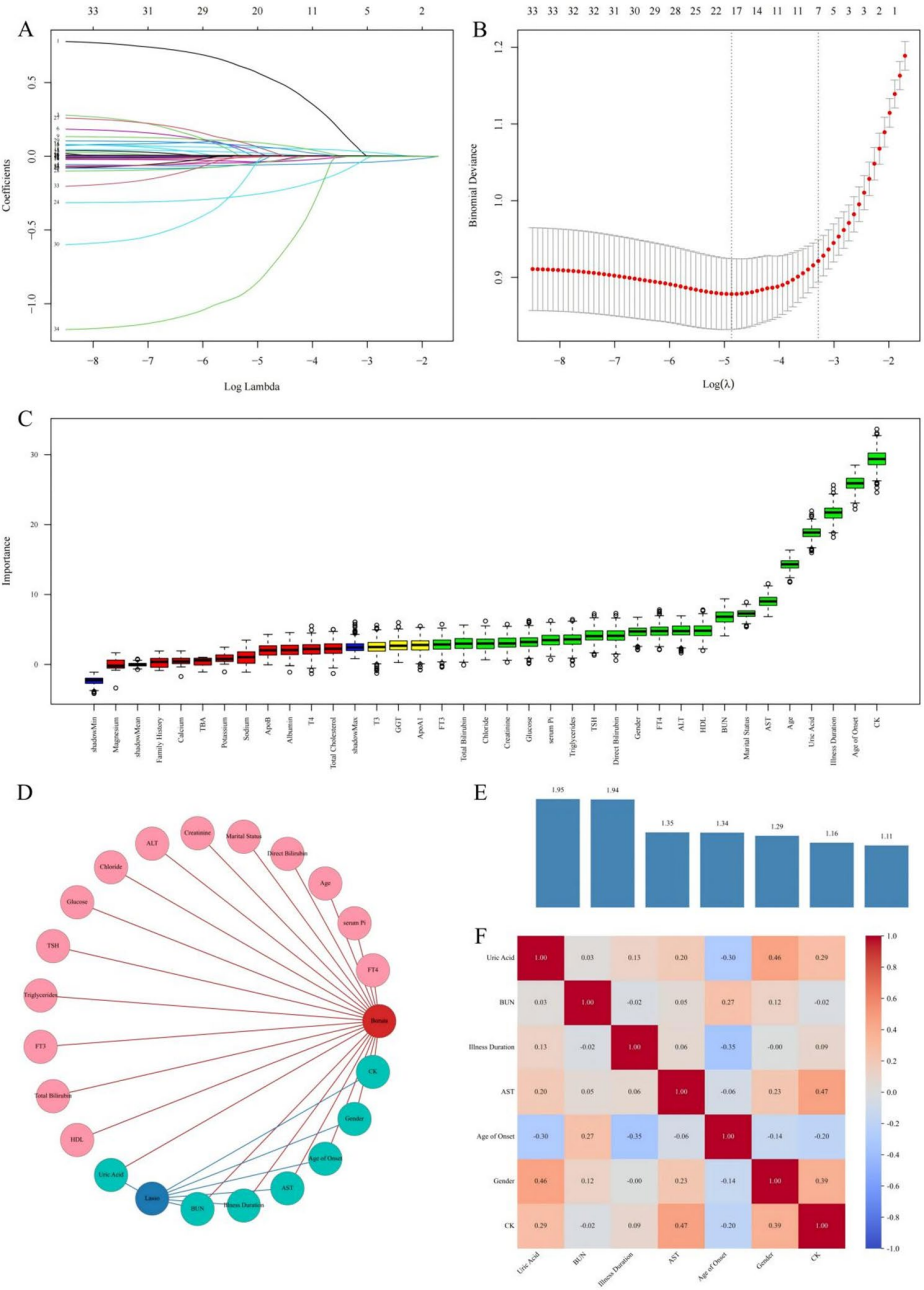
Feature Selection and Analysis. Note: (**A**) LASSO coefficient profiles of the 34 candidate features. (**B**) Selection of the optimal λ parameter via 10-fold cross-validation. Dotted vertical lines indicate the minimum mean error (*λ*_*min*_) and the 1-standard-error rule (*λ*_*1se*_), the latter of which was adopted for this study. (**C**) Feature importance ranking using the Boruta algorithm. Green boxplots denote confirmed important features, red boxplots indicate rejected features, and blue boxplots represent shadow features used as significance thresholds. (**D**) Network visualization of feature selection results. The graph displays the relationship between selection algorithms (Nodes: “LASSO” in blue, “Boruta” in red) and features. (**E**) Variance Inflation Factor (VIF) analysis; all features exhibited VIF values < 2.5, indicating the absence of severe multicollinearity. (**F**) Pearson correlation heatmap of the selected predictors, demonstrating that all pairwise correlation coefficients were below 0.7

Pearson correlation analysis and VIF testing were conducted on the 7 selected features using the development cohort data only. The results demonstrated that all correlation coefficients between features were below 0.7, and all VIF values were below 2.5, indicating the absence of severe multicollinearity issues among the predictor variables [22, 23] (Fig. 1).

### Construction and internal validation of ML models

**Fig. 2:**
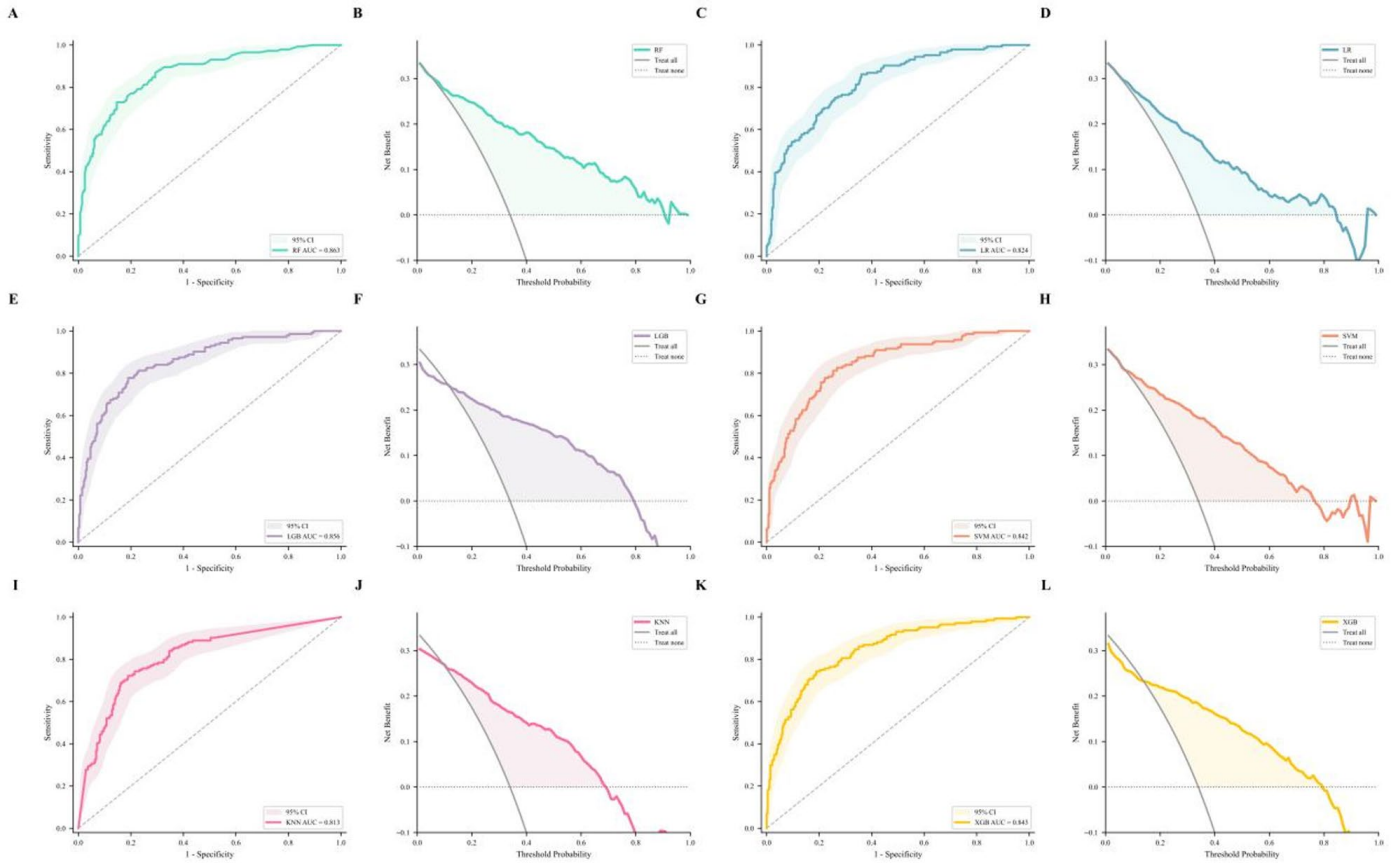
ROC and DCA analysis of six models in internal validation

Six ML algorithms were employed to construct diagnostic models based on the final predictive features. In the internal validation set, the AUC values for RF, LGB, XGB, SVM, LR, and KNN were 0.863, 0.856, 0.843, 0.842, 0.824, and 0.813, respectively (Fig. 2). All models achieved AUC values above 0.8, indicating good discriminatory performance in distinguishing between BD and MDD. Crucially, among all models, the RF model demonstrated the highest AUC of 0.863 in this internal validation set (Table 2).

**Table 2:** Internal validation of model performance

Model	AUC (95% CI)	ACC	SEN	SPE	PPV	NPV	BAC
RF	0.863 (0.825–0.898)	0.807	0.667	0.879	0.738	0.837	0.773
LGB	0.856 (0.814–0.890)	0.804	0.660	0.879	0.736	0.834	0.769
XGB	0.843 (0.801–0.880)	0.785	0.618	0.871	0.712	0.816	0.745
SVM	0.842 (0.801–0.879)	0.781	0.681	0.832	0.676	0.835	0.756
LR	0.824 (0.780–0.861)	0.752	0.681	0.789	0.624	0.828	0.735
KNN	0.813 (0.763–0.851)	0.778	0.715	0.811	0.660	0.847	0.763

Note: RF: Random Forest; LGB: Light Gradient Boosting Machine; XGB: XGBoost; SVM: Support Vector Machine; LR: Logistic Regression; KNN: K-Nearest Neighbors. AUC: Area Under the Receiver Operating Characteristic Curve (95% CI: 95% Confidence Interval calculated via bootstrap); ACC: Accuracy; SEN: Sensitivity; SPE: Specificity; PPV: Positive Predictive Value; NPV: Negative Predictive Value; BAC: Balanced Accuracy

### External validation of the model

**Fig. 3:**
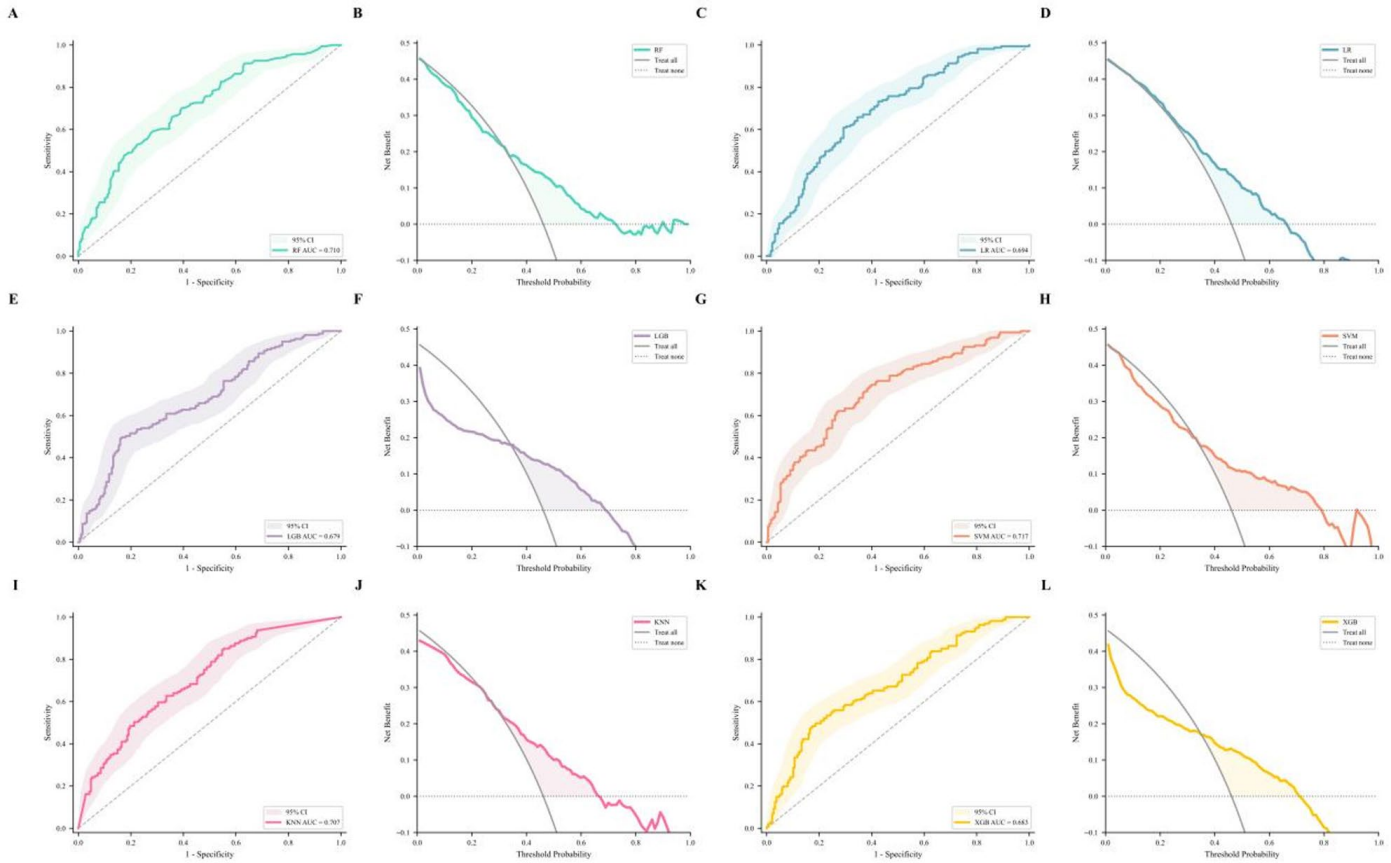
ROC and DCA analysis of six models in external validation

In the external validation cohort, the SVM, RF, KNN, LR, XGB, and LGB models achieved AUC values of 0.717, 0.710, 0.707, 0.694, 0.683, and 0.679, respectively (Fig. 3; Table 3). Both RF and SVM performed well in external validation, with AUCs of 0.710 and 0.717, respectively. After DeLong’s test, there was no statistically significant difference between the two models (*P* = 0.717) (Supplementary Tables S4 and S5). Recognizing the insufficient sensitivity of the RF model at the default threshold of 0.50, we determined an optimal threshold by maximizing the Youden Index within the internal validation set. Applying this optimized threshold to the external validation cohort yielded an improvement in sensitivity (Supplementary Table S6).

**Table 3:** External validation of model performance

Model	AUC (95% CI)	ACC	SEN	SPE	PPV	NPV	BAC
SVM	0.717 (0.662–0.770)	0.645	0.453	0.809	0.670	0.633	0.631
RF	0.710 (0.657–0.767)	0.650	0.422	0.846	0.701	0.631	0.634
KNN	0.707 (0.652–0.763)	0.639	0.596	0.676	0.611	0.661	0.636
LR	0.694 (0.638–0.747)	0.639	0.671	0.612	0.597	0.685	0.641
XGB	0.683 (0.625–0.740)	0.653	0.404	0.867	0.722	0.629	0.635
LGB	0.679 (0.621–0.739)	0.656	0.416	0.862	0.720	0.633	0.639

Note: RF: Random Forest; LGB: Light Gradient Boosting Machine; XGB: XGBoost; SVM: Support Vector Machine; LR: Logistic Regression; KNN: K-Nearest Neighbors. AUC: Area Under the Receiver Operating Characteristic Curve (95% CI: 95% Confidence Interval calculated via bootstrap); ACC: Accuracy; SEN: Sensitivity; SPE: Specificity; PPV: Positive Predictive Value; NPV: Negative Predictive Value; BAC: Balanced Accuracy

### Model interpretability

**Fig. 4:**
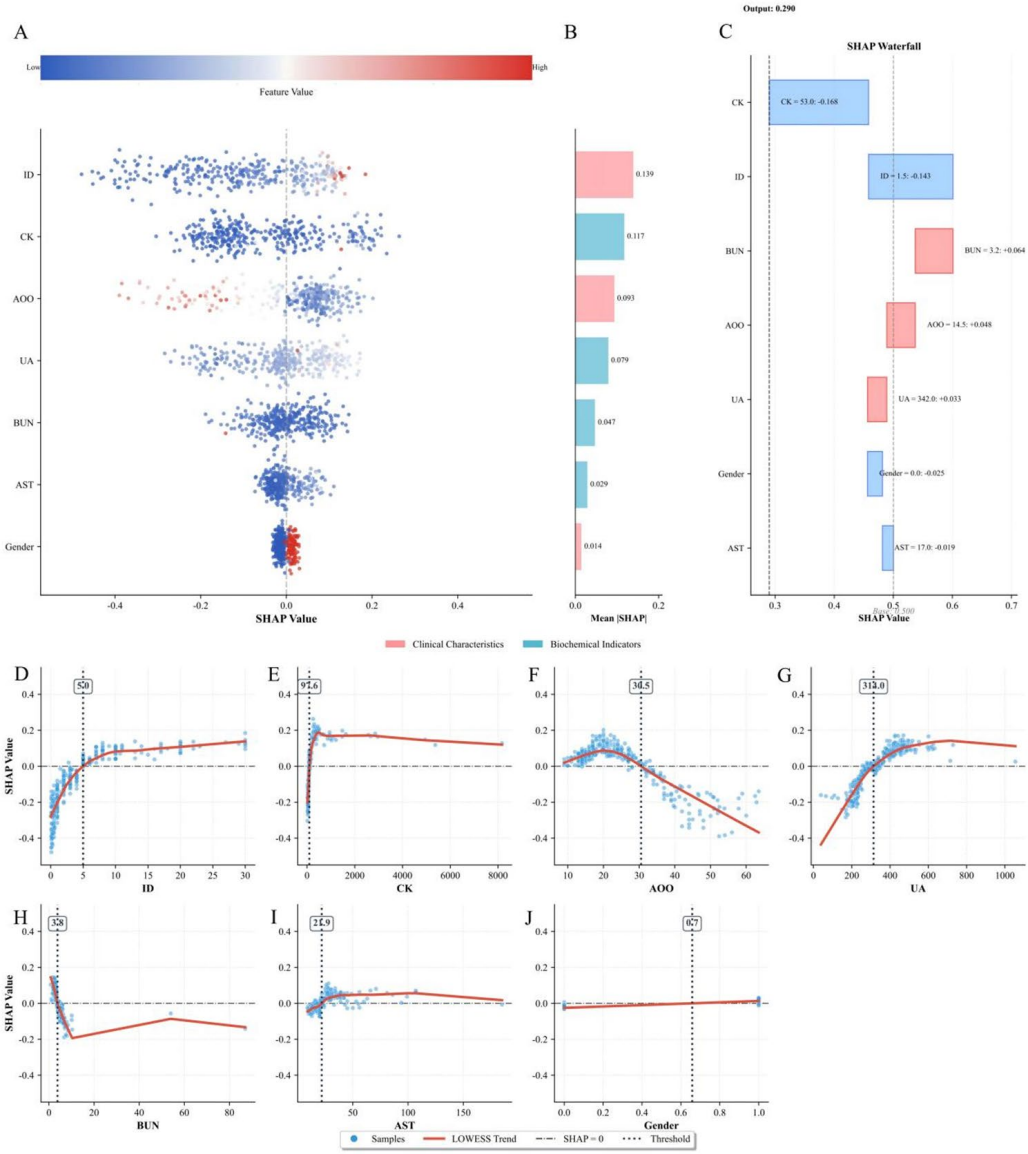
Model Interpretability. Note: (**A**) SHAP beeswarm summary plot. Each dot corresponds to a patient. The x-axis represents the SHAP value, where positive values (SHAP value > 0) signify a contribution to BD prediction and negative values (SHAP value < 0) indicate a tendency toward MDD. Feature magnitude is represented by color (Red = High; Blue = Low). (**B**) SHAP bar plot ranking the 7 features by global importance based on the mean absolute SHAP value. (**C**) Waterfall plot illustrating the prediction decision path for a single representative patient. Red bars indicate features increasing the probability of BD, while blue bars indicate features decreasing it (favoring MDD). (**D**-**J**) SHAP dependence plots for the 7 predictive features, ordered by importance. The x-axis represents the feature value, and the y-axis represents the SHAP value. The red solid line represents the LOWESS fitted trend, and vertical dotted lines mark calculated clinical thresholds where the feature’s contribution shifts between favoring MDD and BD (i.e., crossing the SHAP = 0 line); AOO, Age of Onset; ID, Illness Duration; UA, Uric Acid; CK, Creatine Kinase; BUN, Blood Urea Nitrogen; AST, Aspartate Aminotransferase

The results of the SHAP analysis (Fig. 4A) show that the Y-axis indicates the importance of features in making predictions in descending order, while the X-axis represents the SHAP value. Dots on the right (SHAP value > 0) indicate that patients with feature values contribute to the ‘1’ category (BD) decision, whereas dots on the left (SHAP value ≤ 0) indicate that patients with feature values contribute to the ‘0’ category (MDD) decision. The colour gradient from blue to red represents the feature value, ranging from low to high. SHAP analysis identified illness duration (0.139) as the most important feature, followed by CK (0.117), AOO (0.093), UA (0.079), and BUN (0.047), with AST (0.029) and gender (0.014) contributing less (Fig. 4B).

The single-sample force plot shows a baseline SHAP value of 0.500. The model output value for a single sample is 0.290. In this specific sample, UA and AOO were identified as features contributing positively to the BD prediction, with SHAP values of + 0.033 and + 0.048, respectively. Conversely, illness duration and CK were found to have a negative influence, with SHAP values of -0.143 and − 0.168, respectively (Fig. 4C).

In the external validation set, SHAP dependence plots revealed the impact of individual features on model predictions. Elevated AOO (Fig. 4F) and BUN levels (Fig. 4H) were associated with lower SHAP values, indicating a propensity toward MDD classification. Conversely, higher CK levels (Fig. 4E) and UA levels (Fig. 4G) tended to favor BD classification. Notably, CK values exceeding 94 U/L (Fig. 4E) and illness duration beyond 5 years (Fig. 4D) consistently yielded positive SHAP values (SHAP value > 0), suggesting that exceeding these thresholds drives the model toward a BD classification.

## Discussion

This study aims to develop and validate a clinical diagnostic model to effectively distinguish MDD from BD, facilitating early screening and intervention. From the 34 candidate variables, seven predictive features were determined using LASSO and Boruta algorithm: gender, AOO, illness duration, AST, BUN, UA, and CK. Among the six machine learning models constructed based on these features, the RF model demonstrated the best overall performance, achieving the highest (AUC = 0.863) in the internal validation set and also showing moderate discriminative ability (AUC = 0.710) in the external validation set.

Notably, the AUC of the RF model in the external validation decreased by 0.153 (from 0.863 to 0.710) compared with the internal validation. This performance decay was mainly attributed to domain shift between cohorts and cross-cohort instability of key predictors. There were substantial differences in demographic characteristics between the external validation cohort and the development cohort. Compared to the development cohort, patients in the external validation cohort were younger (30.2 ± 13.7 years vs. 39.8 ± 14.5 years), had an earlier AOO (24.9 ± 11.8 years vs. 33.0 ± 14.5 years), and had a shorter illness duration (5.1 ± 6.3 years vs. 7.2 ± 8.4 years). The proportion of patients with BD was significantly higher (46.1% vs. 29.9%, *P* < 0.001). Some biochemical indicators also showed significant differences between the cohorts. Previous studies have shown that such distribution differences can significantly limit the generalization ability of machine learning models [24].

More importantly, the differences in data distribution suggest that the two cohorts may represent different clinical subtypes, which directly affects the stability of key predictors among different cohorts. For instance, CK can effectively distinguish between MDD and BD in the development cohort (*P* < 0.001), but completely loses the ability to distinguish in the external validation cohort (adjusted *P* = 0.968). However, SHAP analysis shows that the model still regards CK as the second most important feature. The model still highly relies on this feature that is not prominent in the external environment, which directly limits its generalization ability. Nevertheless, the external validation AUC of 0.710 met the acceptable discriminant power criterion (AUC > 0.70), suggesting that the model retains potential as a diagnostic support tool [25, 26].

Current studies distinguishing MDD from BD primarily focus on genetics, sleep studies, and peripheral inflammation to identify biomarkers for differential diagnosis [27–30]. In this study, different features and biomarkers extracted from multiple domains in the EMR system were used to construct a diagnostic model, and its generalization ability was confirmed through rigorous external validation, which provides insights into the biological basis for the stratification and individualized treatment of mood disorders.

Unlike previous studies, this study included rigorous external validation [5, 18, 19]. The ML models constructed by Zhu et al. [18] and Huang et al. [19] based on EMR data performed well, with AUC of 0.838 and 0.858, respectively, but both lacked external validation to verify their generalization ability. In contrast, the RF model constructed in this study performed well in internal validation (AUC = 0.863) and independent external validation (AUC = 0.710), indicating that the model had clinical application potential.

The findings revealed that AOO and illness duration played key roles in distinguishing MDD from BD, with illness duration being the most critical discriminator. This result is consistent with previous literature reports, suggesting the potential value of these two indicators in clinical discrimination [30–33]. Previous studies have confirmed the clinical value of AOO in differentiating BD from MDD [33]. Compared with MDD, the onset age of BD is about 7.5 years earlier on average [33], and this early onset feature naturally leads to a longer illness duration in BD patients. In addition, substantial evidence suggests that a substantial proportion of patients initially diagnosed with MDD convert to BD during long-term follow-up [32, 34, 35]. This conversion is often due to the fact that BD often begins with depression, whereas manic symptoms occur with a lag and take a longer time to appear. These clinical features together support the diagnostic value of AOO and illness duration in the differential diagnosis.

The findings revealed that CK played a key role in distinguishing MDD from BD, which was consistent with the conclusions of Zhu et al., who emphasized the predictive value of CK [18]. Previous studies have shown that CK levels are significantly elevated in BD patients during manic episodes compared to MDD patients. This difference may be attributed to increased physical activity during mania, which accelerates skeletal muscle metabolism and subsequently leads to elevated serum CK levels [36].

Several studies have identified UA as a potential biomarker to distinguish BD from MDD [37–39]. As a product of purine metabolism, UA is closely related to physiological processes such as sleep, exercise, cognitive function and emotional regulation [38]. The present study found that the UA level in BD patients was significantly higher than that in MDD patients, a result consistent with previous literature reports [39].

Epidemiological studies indicate a significant association between impaired renal function and certain psychiatric disorders, particularly BD and MDD [40–42]. Yu et al. identified genetic associations and shared genetic variants between renal function biomarker profiles and BD and MDD at the genome-wide scale [42]. BUN shares a pathogenic variant site with MDD and BD and has been identified as an independent risk factor for psychiatric disorders. However, whether the relationship between BUN and mental disorders is merely a predictive marker or involves participation in the pathological process of the disease remains unclear and requires further longitudinal and mechanistic studies to clarify this relationship.

This study has several limitations. First, the clinical diagnosis is based on the ICD-10 standard rather than using the Fifth Edition of the Diagnostic and Statistical Manual of Mental Disorders (DSM-5), which may lead to some patients with latent BD being wrongly classified as MDD [43]. This introduces label noise and misclassification bias, which could subsequently compromise the model’s predictive accuracy and generalizability. Second, this study only included inpatients in the acute phase without mental comorbidities (e.g., anxiety disorders). Given the prevalence of clinical comorbidities and their potential to interfere with biomarker levels, the effectiveness of this model in outpatient, mild and complex cases still needs further verification. Additionally, our study did not include symptom severity measures, limiting our ability to assess sample heterogeneity in illness severity or mood states. This prevents us from determining whether identified biomarkers represent stable trait markers or state-dependent features, and whether patients in our sample belong to similar clinical subgroups in terms of illness severity and mood states. Finally, differences in the distribution of demographic and clinical characteristics between the external validation cohort and the development cohort suggest that the two may represent different clinical subgroups, and such differences in data distribution may limit the ability of the model to generalize. Future studies should include international multi-center samples to verify the robustness of the model and develop more intuitive decision support tools to enhance its clinical utility.

## Conclusion

The study used EMR data, and LASSO and Boruta methods were employed to select the top 7 features used to distinguish BD from MDD, including gender, AOO, illness duration, AST, BUN, UA, and CK. The RF model consistently achieved the best discriminative performance across datasets. It demonstrated a robust internal validation AUC of 0.863 and an independent external validation AUC of 0.710, indicating moderate generalizability with potential for clinical application as an adjunctive decision-support tool. SHAP analysis further revealed the significant contributions of illness duration, CK, and AOO to model prediction. Although this model achieves moderate discriminatory performance, its current application is limited to hospitalized BD and MDD patients and requires validation across diverse populations and healthcare systems. Future research should incorporate international multicenter samples to validate the models’ generalization capabilities, and develop more intuitive decision support tools to enhance clinical utility. These findings provide preliminary evidence for the auxiliary differential diagnosis of BD and MDD in hospitalized patients.

## Electronic supplementary material

Below is the link to the electronic supplementary material.

Supplementary Material 1

## Data Availability

The datasets generated in this study are available from the corresponding author on reasonable request.
