# Supplementary material for: Development and external validation of a diagnostic model for differentiating major depressive disorder from bipolar disorder
Source: BMC Psychiatry. 2026 Jan 28;26:197. doi: 10.1186/s12888-026-07844-1 (PMC12924540; doi:10.1186/s12888-026-07844-1)
Supplement: Supplementary file 1 — Supplementary Material 1 [file 12888_2026_7844_MOESM1_ESM.docx]

**Supplementary materials**

**Supplementary Table S1.** Hyperparameter search space and optimal values for machine learning models.

| Model | Hyperparameter | Search Space | Optimal Value |
| --- | --- | --- | --- |
| RF | n_estimators | 100, 200 | 200 |
|  | max_depth | None, 10, 20 | None |
|  | min_samples_split | 2, 5 | 2 |
|  | min_samples_leaf | 1, 2 | 1 |
| LR | C | 0.01, 0.1, 1, 10 | 10 |
|  | penalty | l1, l2 | l1 |
| LGB | n_estimators | 50, 100, 200 | 100 |
|  | learning_rate | 0.01, 0.05, 0.1 | 0.1 |
|  | num_leaves | 31, 50 | 50 |
|  | reg_lambda | 0, 0.1 | 0 |
| SVM | C | 0.1, 1, 10 | 10 |
|  | kernel | linear, rbf | rbf |
|  | gamma | scale, auto | scale |
| KNN | n_neighbors | 3, 5, 7, 9 | 7 |
|  | weights | uniform, distance | distance |
| XGB | n_estimators | 100, 200 | 200 |
|  | learning_rate | 0.01, 0.1 | 0.1 |
|  | max_depth | 3, 6 | 6 |

**Note:** Optimal hyperparameters were identified using GridSearchCV with 10-fold stratified cross-validation, optimizing for AUC. "None" indicates no constraint. RF: Random Forest; LR: Logistic Regression; LGB: LightGBM; SVM: Support Vector Machine; KNN: K-Nearest Neighbors; XGB: XGBoost.

**Supplementary Table S2.** Comparison of Baseline Characteristics Between MDD and BD Patients in the Development Cohort

|  | MDD (*n* = 991) | BD (*n* = 423) | (*χ²*/*t*) | *P* | Adjusted *P* |
| --- | --- | --- | --- | --- | --- |
| Gender |  |  | 55.208 | < 0.001 | < 0.001 |
| Female | 665 (67.1%) | 194 (45.9%) |  |  |  |
| Male | 326 (32.9%) | 229 (54.1%) |  |  |  |
| Marital Status |  |  | 54.510 | < 0.001 | < 0.001 |
| Unmarried | 259 (26.1%) | 196 (46.3%) |  |  |  |
| Married | 732 (73.9%) | 227 (53.7%) |  |  |  |
| Family History |  |  | 6.275 | 0.012 | 0.024 |
| No | 924 (93.2%) | 377 (89.1%) |  |  |  |
| Yes | 67 (6.8%) | 46 (10.9%) |  |  |  |
| Age | 42.158 ± 14.756 | 34.284 ± 12.203 | -9.656 | < 0.001 | < 0.001 |
| Age of Onset | 36.756 ± 14.684 | 24.155 ± 9.549 | -16.243 | < 0.001 | < 0.001 |
| Illness Duration | 5.629 ± 7.322 | 10.886 ± 9.396 | 11.318 | < 0.001 | < 0.001 |
| T3 | 1.389 ± 0.474 | 1.458 ± 0.439 | 2.530 | 0.012 | 0.024 |
| T4 | 56.124 ± 50.676 | 53.463 ± 51.682 | -0.899 | 0.369 | 0.448 |
| TSH | 2.163 ± 1.859 | 2.306 ± 1.948 | 1.303 | 0.193 | 0.285 |
| FT3 | 4.110 ± 1.332 | 4.250 ± 1.202 | 1.857 | 0.063 | 0.103 |
| FT4 | 8.663 ± 7.523 | 8.362 ± 7.611 | -0.686 | 0.493 | 0.578 |
| Total Bilirubin | 11.741 ± 6.479 | 11.353 ± 6.939 | -1.008 | 0.314 | 0.400 |
| Direct Bilirubin | 4.715 ± 2.554 | 4.699 ± 2.850 | -0.104 | 0.917 | 0.917 |
| Albumin | 42.442 ± 4.173 | 42.676 ± 3.564 | 1.006 | 0.314 | 0.400 |
| ALT | 20.011 ± 20.025 | 25.909 ± 28.415 | 4.439 | < 0.001 | < 0.001 |
| AST | 18.331 ± 14.132 | 23.700 ± 16.331 | 6.230 | < 0.001 | < 0.001 |
| GGT | 24.162 ± 27.090 | 27.469 ± 28.838 | 2.059 | 0.04 | 0.071 |
| TBA | 3.961 ± 3.472 | 4.380 ± 3.637 | 2.041 | 0.041 | 0.071 |
| Total Cholesterol | 4.393 ± 1.040 | 4.176 ± 0.943 | -3.682 | < 0.001 | 0.001 |
| HDL | 1.310 ± 0.441 | 1.211 ± 0.349 | -4.091 | < 0.001 | < 0.001 |
| Triglycerides | 1.382 ± 1.042 | 1.393 ± 0.882 | 0.194 | 0.846 | 0.872 |
| ApoA1 | 1.295 ± 0.517 | 1.246 ± 0.299 | -1.835 | 0.067 | 0.103 |
| ApoB | 0.817 ± 0.440 | 0.790 ± 0.309 | -1.123 | 0.262 | 0.371 |
| BUN | 4.593 ± 1.560 | 4.036 ± 1.455 | -6.270 | < 0.001 | < 0.001 |
| Creatinine | 62.227 ± 17.665 | 64.643 ± 14.569 | 2.476 | 0.013 | 0.025 |
| Uric Acid | 286.303 ± 92.817 | 361.146 ± 113.598 | 12.953 | < 0.001 | < 0.001 |
| Potassium | 3.886 ± 0.378 | 3.983 ± 0.353 | 4.522 | < 0.001 | < 0.001 |
| Sodium | 141.732 ± 3.084 | 141.554 ± 3.013 | -1.00 | 0.317 | 0.400 |
| Chloride | 105.175 ± 2.810 | 105.648 ± 2.863 | 2.878 | 0.004 | 0.009 |
| Calcium | 2.325 ± 0.222 | 2.319 ± 0.167 | -0.449 | 0.654 | 0.694 |
| CK | 117.817 ± 430.392 | 333.100 ± 595.187 | 7.628 | < 0.001 | < 0.001 |
| Glucose | 5.166 ± 1.468 | 4.937 ± 0.950 | -2.941 | 0.003 | 0.008 |
| Magnesium | 0.900 ± 0.122 | 0.897 ± 0.085 | -0.474 | 0.636 | 0.694 |
| Serum Pi | 1.203 ± 0.171 | 1.196 ± 0.177 | -0.637 | 0.524 | 0.594 |

**Note:** T3: Triiodothyronine; T4: Tetraiodothyronine; TSH: Thyroid Stimulating Hormone; FT3: Free Triiodothyronine; FT4: Free Thyroxine; ALT: Alanine Aminotransferase; AST: Aspartate Aminotransferase; GGT: Gamma-Glutamyl Transferase; TBA: Total Bile Acid; HDL: High Density Lipoprotein; ApoA1: Apolipoprotein A1; ApoB: Apolipoprotein B; BUN: Blood Urea Nitrogen; CK: Creatine Kinase; Serum Pi: Serum Inorganic Phosphate; Adjusted *P*: P-values were adjusted for multiple comparisons using the Benjamini-Hochberg False Discovery Rate (FDR) method.

**Supplementary Table S3.** Comparison of Baseline Characteristics Between MDD and BD Patients in the External Validation Cohort

| Variable | MDD (*n* = 188) | BD (*n* = 161) | (*χ²*/*t*) | *P* | Adjusted *P* |
| --- | --- | --- | --- | --- | --- |
| Gender |  |  | 9.466 | 0.002 | 0.020 |
| Female | 139 (73.9%) | 93 (57.8%) |  |  |  |
| Male | 49 (26.1%) | 68 (42.2%) |  |  |  |
| Marital Status |  |  | 6.871 | 0.009 | 0.050 |
| Unmarried | 104 (55.3%) | 112 (69.6%) |  |  |  |
| Married | 84 (44.7%) | 49 (30.4%) |  |  |  |
| Family History |  |  | 0.002 | 0.962 | 0.968 |
| No | 173 (92.0%) | 147 (91.3%) |  |  |  |
| Yes | 15 (8.0%) | 14 (8.7%) |  |  |  |
| Age | 31.271 ± 14.479 | 28.882 ± 12.754 | -1.623 | 0.106 | 0.326 |
| Age of Onset | 27.013 ± 13.328 | 22.443 ± 9.091 | -3.679 | < 0.001 | 0.005 |
| Illness Duration | 4.169 ± 5.452 | 6.216 ± 7.095 | 3.043 | 0.003 | 0.021 |
| T3 | 1.408 ± 0.391 | 1.453 ± 0.359 | 1.126 | 0.261 | 0.554 |
| T4 | 114.747 ± 50.336 | 115.106 ± 25.852 | 0.081 | 0.935 | 0.968 |
| TSH | 1.960 ± 2.191 | 1.863 ± 1.310 | -0.492 | 0.623 | 0.921 |
| FT3 | 5.022 ± 1.091 | 5.038 ± 0.701 | 0.159 | 0.874 | 0.968 |
| FT4 | 11.654 ± 3.388 | 11.379 ± 2.425 | -0.859 | 0.391 | 0.700 |
| Total Bilirubin | 12.054 ± 5.908 | 12.721 ± 6.020 | 1.042 | 0.298 | 0.564 |
| Direct Bilirubin | 2.309 ± 1.202 | 2.328 ± 1.121 | 0.152 | 0.879 | 0.968 |
| Albumin | 40.397 ± 3.787 | 41.685 ± 4.409 | 2.933 | 0.004 | 0.024 |
| ALT | 17.570 ± 12.735 | 22.045 ± 21.385 | 2.407 | 0.017 | 0.063 |
| AST | 21.449 ± 17.045 | 23.281 ± 15.439 | 1.042 | 0.298 | 0.564 |
| GGT | 20.766 ± 23.115 | 26.737 ± 21.502 | 2.465 | 0.014 | 0.060 |
| TBA | 5.294 ± 8.990 | 4.227 ± 4.392 | -1.337 | 0.182 | 0.423 |
| Total Cholesterol | 4.344 ± 0.999 | 4.351 ± 0.976 | 0.067 | 0.947 | 0.968 |
| HDL | 1.241 ± 0.349 | 1.226 ± 0.311 | -0.391 | 0.696 | 0.968 |
| Triglycerides | 1.254 ± 0.777 | 1.230 ± 0.789 | -0.274 | 0.784 | 0.968 |
| ApoA1 | 1.340 ± 0.323 | 1.298 ± 0.237 | -1.323 | 0.187 | 0.423 |
| ApoB | 0.796 ± 0.235 | 0.867 ± 0.250 | 2.67 | 0.008 | 0.043 |
| BUN | 4.366 ± 3.899 | 4.688 ± 6.669 | 0.561 | 0.575 | 0.921 |
| Creatinine | 61.786 ± 15.001 | 64.480 ± 16.859 | 1.579 | 0.115 | 0.326 |
| Uric Acid | 304.035 ± 105.509 | 369.836 ± 115.482 | 5.56 | < 0.001 | < 0.001 |
| Potassium | 3.793 ± 0.299 | 3.813 ± 0.331 | 0.598 | 0.551 | 0.921 |
| Sodium | 138.752 ± 2.035 | 138.916 ± 3.830 | 0.505 | 0.614 | 0.921 |
| Chloride | 104.735 ± 2.086 | 104.745 ± 2.351 | 0.04 | 0.968 | 0.968 |
| Calcium | 2.283 ± 0.182 | 2.312 ± 0.107 | 1.806 | 0.072 | 0.244 |
| CK | 209.576 ± 817.016 | 232.109 ± 472.262 | 0.304 | 0.761 | 0.968 |
| Glucose | 4.912 ± 0.999 | 4.921 ± 1.042 | 0.074 | 0.941 | 0.968 |
| Magnesium | 0.887 ± 0.076 | 0.875 ± 0.080 | -1.457 | 0.146 | 0.382 |
| Serum Pi | 1.336 ± 0.199 | 1.343 ± 0.226 | 0.307 | 0.759 | 0.968 |

**Note:** T3: Triiodothyronine; T4: Tetraiodothyronine; TSH: Thyroid Stimulating Hormone; FT3: Free Triiodothyronine; FT4: Free Thyroxine; ALT: Alanine Aminotransferase; AST: Aspartate Aminotransferase; GGT: Gamma-Glutamyl Transferase; TBA: Total Bile Acid; HDL: High Density Lipoprotein; ApoA1: Apolipoprotein A1; ApoB: Apolipoprotein B; BUN: Blood Urea Nitrogen; CK: Creatine Kinase; Serum Pi: Serum Inorganic Phosphate; Adjusted *P*: P-values were adjusted for multiple comparisons using the Benjamini-Hochberg False Discovery Rate (FDR) method.

**Supplementary Table S4.** Pairwise DeLong's test P-value matrix for all models in external validation

| Model | RF | SVM | LGB | XGB | LR | KNN |
| --- | --- | --- | --- | --- | --- | --- |
| RF | — | 0.717 | 0.013* | 0.042* | 0.477 | 0.913 |
| SVM | 0.717 | — | 0.097 | 0.122 | 0.377 | 0.635 |
| LGB | 0.013* | 0.097 | — | 0.685 | 0.575 | 0.267 |
| XGB | 0.042* | 0.122 | 0.685 | — | 0.681 | 0.343 |
| LR | 0.477 | 0.377 | 0.575 | 0.681 | — | 0.595 |
| KNN | 0.913 | 0.635 | 0.267 | 0.343 | 0.595 | — |

**Note:** Values represent P-values from DeLong's test comparing the AUC between each pair of models in the external validation cohort (n = 349). The matrix is symmetric; significant differences indicate that one model has superior discriminative ability over another. RF: Random Forest; LGB: Light Gradient Boosting Machine; XGB: XGBoost; SVM: Support Vector Machine; LR: Logistic Regression; KNN: K-Nearest Neighbors. *p < 0.05.

**Supplementary Table S5.** DeLong's test for pairwise AUC comparison between RF and other models in external validation.

| Comparison | RF AUC (95% CI) | Other AUC (95% CI) | Difference | *P* | Significance |
| --- | --- | --- | --- | --- | --- |
| RF vs SVM | 0.710 (0.657–0.767) | 0.717 (0.662–0.770) | -0.007 | 0.717 | ns |
| RF vs KNN | 0.710 (0.657–0.767) | 0.707 (0.652–0.763) | + 0.003 | 0.913 | ns |
| RF vs LR | 0.710 (0.657–0.767) | 0.694 (0.638–0.747) | + 0.016 | 0.477 | ns |
| RF vs XGB | 0.710 (0.657–0.767) | 0.683 (0.625–0.740) | + 0.027 | 0.042 | * |
| RF vs LGB | 0.710 (0.657–0.767) | 0.679 (0.621–0.739) | + 0.031 | 0.013 | * |

**Note:** DeLong's test was used to compare the AUC between models in the external validation cohort; 95% CI was calculated using 1,000 bootstrap resamples. RF: Random Forest; LGB: Light Gradient Boosting Machine; XGB: XGBoost; SVM: Support Vector Machine; LR: Logistic Regression; KNN: K-Nearest Neighbors; CI: confidence interval; ns: not significant. **p* < 0.05.

**Supplementary Table S6.** Comparison of diagnostic performance for the Random Forest model using default versus optimized thresholds

| Dataset | Threshold | AUC | SEN | SPE | PPV | NPV | ACC | BAC | Youden Index |
| --- | --- | --- | --- | --- | --- | --- | --- | --- | --- |
| Internal Validation | 0.50 | 0.863 | 0.667 | 0.879 | 0.738 | 0.837 | 0.807 | 0.773 | 0.545 |
|  | 0.40* | 0.863 | 0.729 | 0.854 | 0.719 | 0.860 | 0.811 | 0.791 | 0.583 |
| External Validation | 0.50 | 0.710 | 0.422 | 0.846 | 0.701 | 0.631 | 0.650 | 0.634 | 0.268 |
|  | 0.40* | 0.710 | 0.528 | 0.777 | 0.669 | 0.658 | 0.662 | 0.652 | 0.305 |

**Note:** The optimal threshold (0.40) was determined by maximizing the Youden Index in the internal validation set and subsequently applied to the external validation set; ACC, Accuracy; AUC, Area Under the Curve; BAC, Balanced Accuracy; NPV, Negative Predictive Value; PPV, Positive Predictive Value; SEN, Sensitivity; SPE, Specificity.
